# Biomechanical Influences on Mesh-Related Complications in Incisional Hernia Repair

**DOI:** 10.3389/fsurg.2021.763957

**Published:** 2021-10-29

**Authors:** Friedrich Kallinowski, Yannique Ludwig, Dominik Gutjahr, Christian Gerhard, Hannah Schulte-Hörmann, Lena Krimmel, Carolin Lesch, Katharina Uhr, Philipp Lösel, Samuel Voß, Vincent Heuveline, Matthias Vollmer, Johannes Görich, Regine Nessel

**Affiliations:** ^1^General, Visceral and Transplantation Surgery, University Hospital Heidelberg, Heidelberg, Germany; ^2^Engineering Mathematics and Computing Lab (EMCL), Interdisciplinary Center for Scientific Computing, Heidelberg, Germany; ^3^Laboratory of Fluid Dynamics and Technical Flows, Otto-von-Guericke University Magdeburg, Magdeburg, Germany; ^4^Biomechanics, Hamburg University of Technology, Hamburg, Germany; ^5^Radiological Center, Eberbach, Germany; ^6^General, Visceral and Pediatric Surgery, Klinikum Am Gesundbrunnen, Heilbronn, Germany

**Keywords:** bench test, computerized tomography, incisional hernia, GRIP, CRIP, hernia repair

## Abstract

**Aim:** Hernia repair strengthens the abdominal wall with a textile mesh. Recurrence and pain indicate weak bonds between mesh and tissue. It remains a question which biomechanical factors strengthen the mesh-tissue interface, and whether surgeons can enhance the bond between mesh and tissue.

**Material and Methods:** This study assessed the strength of the mesh-tissue interface by dynamic loads. A self-built bench test delivered dynamic impacts. The test simulated coughing. Porcine and bovine tissue were used for the bench test. Tissue quality, mesh adhesiveness, and fixation intensity influenced the retention power. The influences were condensed in a formula to assess the durability of the repair. The formula was applied to clinical work. The relative strength of reconstruction was related to the individual human abdominal wall. From computerized tomography at rest and during Valsalva's Maneuver, the tissue quality of the individual patient was determined before surgery.

**Results:** The results showed that biomechanical parameters observed in porcine, bovine, and human tissue were in the same range. Tissues failed in distinct patterns. Sutures slackened or burst at vulnerable points. Both the load duration and the peak load increased destruction. Stress concentrations elevated failure rates. Regional areas of force contortions increased stress concentrations. Hernia repair improved strain levels. Measures for improvement included the closure of the defect, use of higher dynamic intermittent strain (DIS) class meshes, increased mesh overlap, and additional fixation. Surgeons chose the safety margin of the reconstruction as desired.

**Conclusion:** The tissue quality has now been introduced into the concept of a critical and a gained resistance toward pressure-related impacts. A durable hernia repair could be designed from available coefficients. Using biomechanical principles, surgeons could minimize pain levels. Mesh-related complications such as hernia recurrence can potentially be avoided in incisional hernia repair.

## Introduction

Incisional hernia is unwanted, but a frequent side-effect of major surgery. Despite the augmentation of the abdominal wall with a textile mesh, incisional hernia recurs frequently. Troubleshooting must analyze not just the meshes, but tissues, fixation techniques, and the strength of the respective interfaces. Recurrence indicates a bond between tissue and mesh too weak to withstand physical activity durably. The weak bond is mechanically overloaded early (1). Healing is impaired since non-crosslinked collagen stretches and results in occult fascial dehiscence (2). A continuously overburdened healing process manifests at a later stage as a recurrent hernia defect (3, 4). A recurrent hernia doubles the utility cost compared with an uncomplicated primary repair (5).

In the planning of an incisional hernia repair, surgeons seek to design a reconstruction that will reach a safe steady-state called a shakedown. The minimal relative motion of the repair materials and the nearby tissues will permit healing and prevent pain, seroma formation, and recurrences. There is a fundamental gap in understanding the degree to which a mechanical mismatch between hernia repair materials and host tissue contributes to failure (6). Therefore, it remains a question whether critical influences can be identified for tissues, meshes, fixation techniques, and interfaces, and whether critical elements can be empowered to withstand destructive forces. Regarding this, there is a need to investigate what are the critical stresses to be withstood. Furthermore, inquiries stand which stress states must necessarily be taken into account for a long-lasting repair, and where are the areas of particular interest, such as the edges, the overlap, the defect closure zone, or the unstable abdominal wall, to name a few.

A model system was developed with porcine and bovine tissues. In order to get both a load case and an assessment of durability, a bench test was built. The bench test delivers cyclic load similar to coughs (7). Meshes and fixation materials were investigated. In order to apply these factors to incisional hernia repair in humans, individual human abdominal walls were analyzed before treatment using computed tomography of the abdomen at rest and during Valsalva's maneuver (9, 30, 31). Relationships were found between important influences on mechanical stability and the repair needed by the individual patient (8, 9).

The aim of the study was to identify the relevant biomechanical influences, such as the tissue quality, load-bearing capacity, and load limit. Repairs that take these influences into account result in load-bearing abdominal walls and reduce mesh-related complications. Our knowledge is limited which specific influences are critical. Since pulse load studies on animal and human tissues with subsequent application to clinical incisional hernia repair are rare, further studies are needed to fully explore the potential of this promising new approach.

## Materials and Methods

### Tissue Testing With the Texture Analyser®

The Texture Analyser® uses uniaxial disruption to characterize tissue samples (TA.XT *plus*; Stable Micro Systems, Godalming, UK) at a tracking speed of at least 10 mm/min^−1^ without a force limit. The experiments with the Texture Analyzer® (TA) were conducted at the Institute of Pharmacology and Molecular Biotechnology of Heidelberg University (IPMB). In general, TA is used in pharmaceutical, food, or cosmetical industries to analyze the hardness, failure load, or fluidity of a product. In this study, tissue samples derived from the punched defect were tested. The tissue samples were collected, retained frozen, and defrosted 24 h before running the test. Stress and strain were measured until the failure of the sample. One half of each sample group was prepared longitudinally, the other half crosswise to the linea alba of the porcine abdomen and the muscle fiber direction of the bovine flank respectively. The stiffness of the samples was evaluated using the steepest part of the incline of the stress-strain curve. The Young modulus was calculated as an increase in force per square millimeter necessary for the respective strain.

### The Bench Test for Dynamic Intermittent Strain (DIS)

The bench test was self-built using a stainless-steel water reservoir delivering pressure peaks repeatedly under computer control on a plastic foil (9, 30). The bovine or porcine tissue was mounted above the foil without tension applying a baseline pressure above 4 and below 10 mmHg. The bench test repeatedly delivers pressure peaks with a plateau phase. The plateau phase can be varied to change the duration of the peak pressure and permits variable energy transfer. For the experiments described here, central defects of the tissue were standardized with a punch. Closure of the defect and reinforcement of the suture line or bridging with various hernia meshes was performed as desired. The mesh was placed between the muscle and the fascia or the peritoneum in the so-called sublay position or onlay on top of the musculature or below the peritoneum or fascia in an underlay fashion. Dynamic intermittent strain (DIS) was applied with peak pressures of 120 to 250 mmHg according to protocol.

Knowledge of the critical and the gained resistance to impacts related to pressure (the GRIP concept) enables the evaluation of the bonding strength of compound fixations (8). Two sets of experiments were conducted. First, three series used unsecured meshes bridging 5 and 7.5 cm defects in porcine and bovine tissues. Secondly, meshes were secured with tackers. Each group consisted of 10 experiments for the DIS model. The use of 7.5 cm hernia orifices covered with 15 × 15 cm meshes aims at the assessment of reduced stability. Since fixation was needed to reach adequate GRIP values, the meshes were secured with 16 single point fixations of two different staplers, using either Securestrap® (Ethicon, Norderstedt, Germany) or AbsorbaTack® (Medtronic, Meerbusch, Germany). The compounds were loaded up to 425 times with cyclic impacts up to 220 mmHg.

### Analysis of Human Tissue Distension *in vivo*

The properties of human abdominal walls were investigated for comparison to animal tissue samples. A total of 110 patients with ventral incisional hernias [66 men, 44 women; mean (± SD) age: 62 ± 12 years] were subjected to computed tomography of the abdomen at rest and during Valsalva's maneuver. The scans were done preoperatively in a low-dose technique without a contrast agent. A standardized characterization of the hernia and the abdominal wall were performed according to previously published procedures (9, 10, 31). The regional distribution of stress and strain in the human abdominal wall was analyzed with imaging techniques using self-developed artificial intelligence and non-rigid b-splines applied to CT scans of the abdomen at rest and during Valsalva's maneuver. The resolution of the resulting strain distribution was about 1 cm (11). From the picture analysis, the tissue distension and the subsequent strain distribution as changes in length per measured distance were obtained (see **Figure 8** for illustration).

### Influence of the Loading Stress

Load-limit curves were obtained from bovine flancs punched with a 5 cm round defect. The defect was covered with a square Dynamesh® CiCAT 15 × 15 cm in the sublay position without fixation (DynaMesh® Cicat, FEG Textiltechnik, Aachen, Germany). In the first series, the length of the plateau phase was increased. Durations of 0.1, 0.2, and 0.4 s were used. In a second series, the peak pressure was increased from 120 to 150, then to 180 and 210 mmHg at constant plateau lengths of 0.1 s. Under each condition, the experiments were repeated 10 times with fresh preparations tested up to 425 repeated impacts or mesh dislocation if this occurred earlier.

### Influence of the Defect Closure Technique and the Resulting Stress Concentration

Most surgical textbooks advise the resection of the hernia sac and the closure of the defect using a tension-free repair. Since the excision of a scar or a hernia sac decreases the amount of tissue available for closure, the radius of the abdominal curvature is reduced leading to tension. The simulation of this process requires standardized defects with similar areas. As a first approach, defects of 19.6 to 20.13 cm^2^ were created using round or rhomboid punches with a diameter of 5 cm or width of 3.5 cm and a length of 12.1 cm. Porcine abdominal walls were used as a tissue for this investigation. The rhomboid defects were oriented longitudinally with the umbilical area as the center. The round defects were centered around the umbilicus. The defects were covered with round Dynamesh® Cicat meshes with a diameter of 12.5 cm without fixation or were sutured with MonoMax® size 2-0 HR 37 needle in a small-bite-technique or using large bites for closure. Monomax® is an ultra-long-term tear-resistant, resorbable, elastic, monofilament suture made of poly-4-hydroxybutyrate (B. Braun, Aesculap, Tuttlingen, Germany). The suture-to-incision ratio was 7.1–7.2 for the round defects and 6.1–6.2 for the rhomboid-shaped orifices.

The transfer of these data to the human situation requires the analysis of the spatial strain distribution within the abdominal wall. For this purpose, CT scans of the abdomen at rest and during Valsalva's maneuver were studied in an academic teaching hospital, a maximum care surgical unit, and a rural community hospital (9, 31). A total of 110 consecutive patients were studied between June 2018 and December 2020 with at least 160 scans by at least three different observers (10, 11, 31). The pixel size of the reconstructions varied between an edge length of 1 to 10 mm giving a spatial resolution of at least 1 cm.

### Statistical Analysis

Parametrical and non-parametrical data were calculated. Box-Whisker-Plots and likelihood or probability curves for durable repairs were used for the depiction of data. Non-parametric tests were performed with Kruskal-Wallis tests for group homogeneity and Mann-Whitney u-tests to analyze significant differences between two observations.

## Results

The tensile strength of the tissues with the Texture Analyser® and the concomitant retention force assessment with pulse loads upon DIS testing were assessed to gain insight into the influence of the tissue quality on a durable hernia repair.

### Tissue Testing With the Texture Analyser®

**Figure 1:**
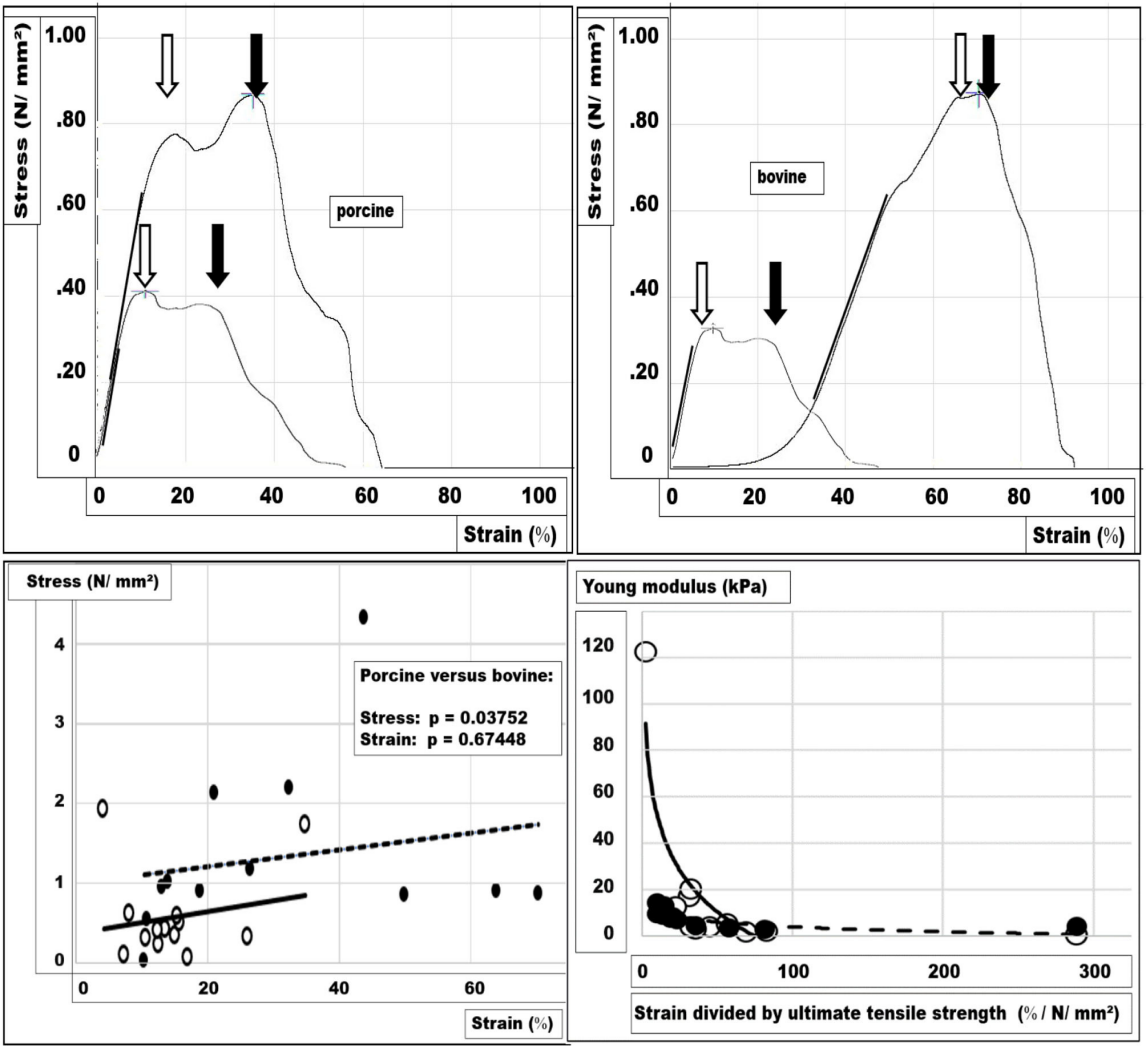
Stress-strain relations were analyzed with the TA. Examples of the stress-strain relations for porcine **(left)** and bovine tissues **(right)** are depicted in the upper panel. In both panels, two peaks are marked with arrows indicating a ductile failure pattern. A cross marks the load-bearing capacity at the higher peak. In addition, the line used for the calculation of the stiffness is indicated on the upper panels as a thicker straight line fitted by eye to the curves. In the lower-left panel, the ultimate tensile strength is given for each sample of both tissues (dots = bovine, circles = porcine) as a function of its respective strain at the breaking point. In the lower right panel, the modules describing the mechanical tissue compliance are depicted for both tissues (dots = bovine, circles = porcine). In the lower panels, the trend lines are indicated for porcine (solid) and bovine (broken) tissue. As a trend, the bovine tissue has more tensile strength, whereas porcine tissue is less distensible **(lower left panel)**. At a higher stiffness, the tissues are less distensible normalized for 1 N/mm^2^. Porcine tissue stretches less when 1 N/mm^2^ stress is applied, whereas bovine tissue is more compliant **(lower right panel)**.

Ultimate tensile stress and tissue elasticity were analyzed obtaining the stress-strain relationship for the animal tissue investigated until failure. Tissue analysis was performed with the Texture Analyser® using 25 tissue samples (12 bovines, 13 porcine). In about half of the cases, a single-cusp curve was recorded (6 bovines, 6 porcine samples). A single-cusp curve indicates that one predominant tensile structure is taking over the load-bearing capacity. In 11 cases, a double-cusp curve was observed (4 bovines, 7 porcine samples) indicating two tensile structures to bear the load. In two bovine samples, an intermediate type occurred. Examples of the stress-strain relations are depicted in Figure 1, upper panel.

For bovine tissue samples, the tensile strength at failure was two and a half times that of porcine tissue samples (mean stress ± SD for bovine tissue = 1.325 ± 1.070 N/mm^2^, for porcine tissue = 0.573 ± 0.607 N/mm^2^). The strain level at the failure of bovine tissue samples was twice that of porcine tissue samples (mean strain ± SD for bovine tissue = 31 ± 20%, for porcine tissue = 15 ± 8%). The differences were not significant at the 1% level for both stress and strain (two-sided u-test: *p* = 0.03752; *p* = 0.67448).

The tissue stiffness is represented by Young‘s modulus, which was taken by fitting a straight line to the incline of the steepest part of the stress-strain curve. Porcine tissue was about two and a half times as stiff as the bovine samples (mean: 0.078 N/mm^2^ vs.0.174 N/ mm^2^; u-test: *p* = 0.01278).

In the lower panels of Figure 1, the stress-strain relationship at the ultimate tensile strength and the stiffness at a normalized tensile strength is depicted. Distinct differences between the tissues and the marked interindividual variation can be seen.

### Bench Tests for Dynamic Intermittent Strain (DIS)

**Figure 2:**
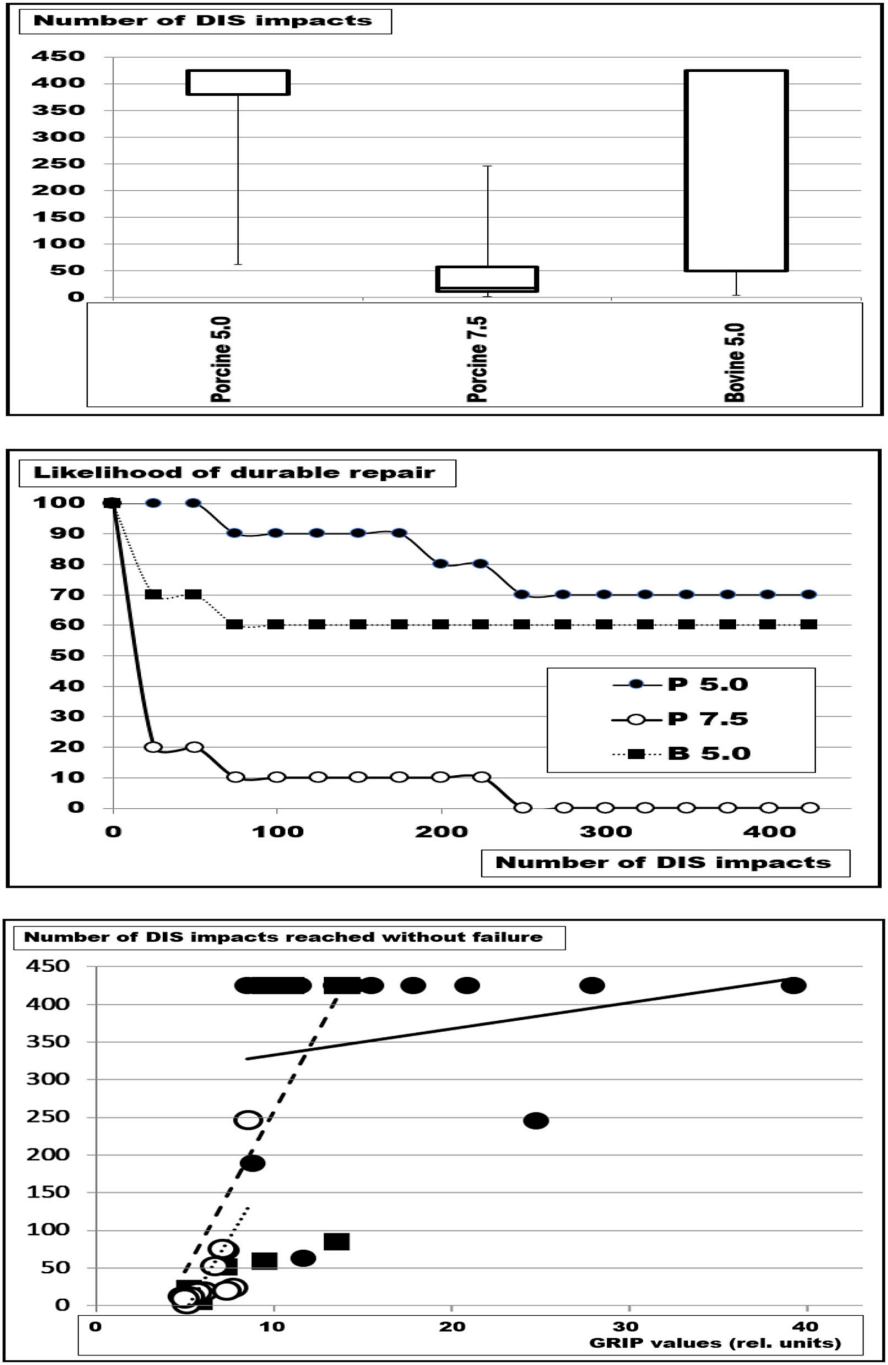
Box-and-whisker plots **(top)**, the likelihood of durable repairs **(middle)**, and the number of DIS impacts without failure as a function of the GRIP values adjusted to the distension of the orifice **(bottom)**. Depicted are the results of bridging of defects with a diameter of 5 cm (5) and 7.5 cm (7.5) in porcine (P), and bovine (B) tissues with square Dynamesh® Cicat meshes 15 cm x 15 cm without fixation. The symbols in the lower panel correspond to those in the middle panel. The trendline in the lower panel for P 5 is solid, that for P 7.5 is dotted and that for B 5 is broken. It can be seen that the trendlines for P 7.5 and B 5 run in parallel. The solid line increases with increasing GRIP reaching a durable repair at GRIP values above 25.

In order to test the influence of tissue quality on fixation requirements, a set of repair designs was assessed (Figure 2). In the first series of experiments, porcine abdominal walls with 5 cm orifices bridged with an unsecured 15 × 15 cm Dynamesh® Cicat mesh showed dislocation in 3 out of 12 samples upon 425 DIS impacts (Figure 2, upper and middle panel). A defect size of 7.5 cm diameter could not be safely bridged with an unsecured 15 × 15 cm Dynamesh® Cicat mesh. Dislocation occurred between two and 246 impacts (median: 18 DIS impacts). The GRIP analysis demonstrated that the increase in hernia diameter decreases the safety of the repair markedly as indicated by the trendlines (Figure 2, lower panel). In bovine tissue, a 5 cm orifice bridged with an unsecured 15 × 15 cm Dynamesh® Cicat, was successfully repaired in two-thirds of the cases (Figure 2, upper panel; median: 425, range: 5–425). The likelihood of a durable repair drops markedly for the first 200 DIS impacts. Thereafter, a stabilization occurs the likelihood of a durable repair being only 10% lower after 425 strokes as compared with the porcine tissue with a 5 cm large hernia (Figure 2, middle panel). The differences were significant with the Kruskal-Wallis test (*p* = 0.00058). The trendline of the bovine tissue with a 5 cm defect runs parallel to that of porcine tissue with a 7.5 cm orifice.

It was concluded that more elastic bovine tissue exhibited increased dislocation rates even after adjustment of the GRIP values to the increase of the distended defect area (Figure 2). Elastic tissue permitted more creep, increased failure, and should be taken into account when a durable hernia repair would be designed.

In a second series, a defect of 7.5 cm was safely bridged with a 15 cm x 15 cm Dynamesh® Cicat when 16 single point fixations of Securestrap® were used. No dislocation occurred. The results were independent of the used tissue (bovine/porcine). Using AbsorbaTack®, a dislocation rate of 50% was observed under the same conditions. The statistical analysis with the u-test was not significant. It is concluded that the load-bearing capacity of the tissue permits durable repairs even at higher tissue elasticity.

### Analysis of Human Tissue Distension *in vivo*

**Figure 3:**
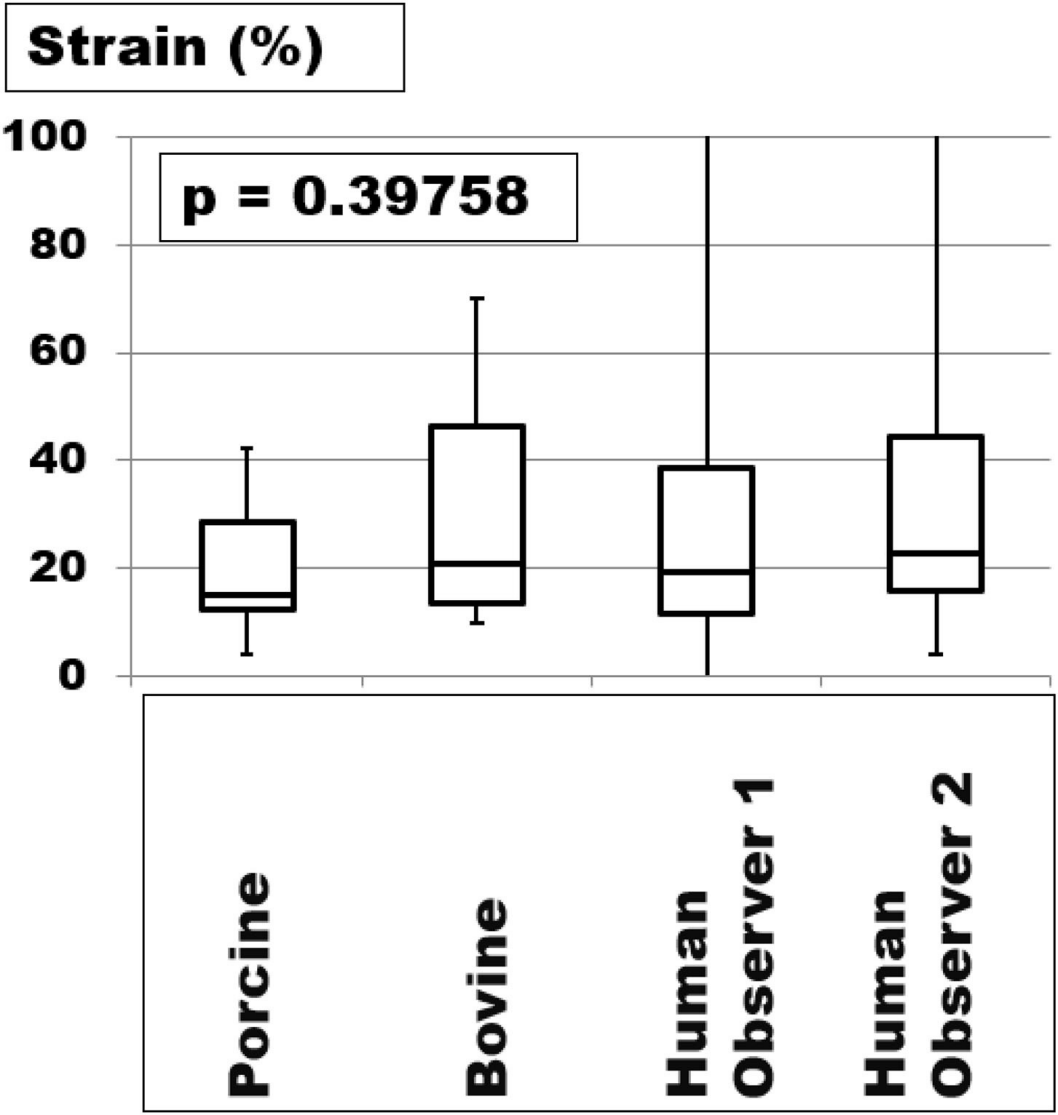
The strain was measured in porcine and bovine samples with the Tissue Analyser® and human tissue with computerized tomography of the abdomen at rest and during Valsalva's maneuver. The box- and whisker plots are the results of at least fifteen up to forty observations. The human tissues vary more with the whiskers up to the maxima being truncated at 100% for the sake of the clarity of the graph.

In order to transfer the results from animal tissue to the human situation, we analyzed the strain distribution in porcine, bovine, and human tissue (Figure 3). In the experimental situation, all tissue preparations were dilated upon pressure. The extent varied from 5 to 42% in porcine abdominal walls and 10 to 72 % in bovine flancs. Herniated human abdominal walls varied much more, up to 18-fold with some abdominal walls staying almost constant upon straining. The contracture of the musculature even decreased the hernia diameter in rare cases. The interobserver variation was high in any given sample. Repeated measurements with an average of 12 observations brought the variation below 5%. On average, the strain levels were comparable in the different species (Kruskal-Wallis test: *p* = 0.39758). We concluded that the insight gained from animal investigations can be applied to the human situation if repeated observations were made in the latter case.

**Figure 4:**
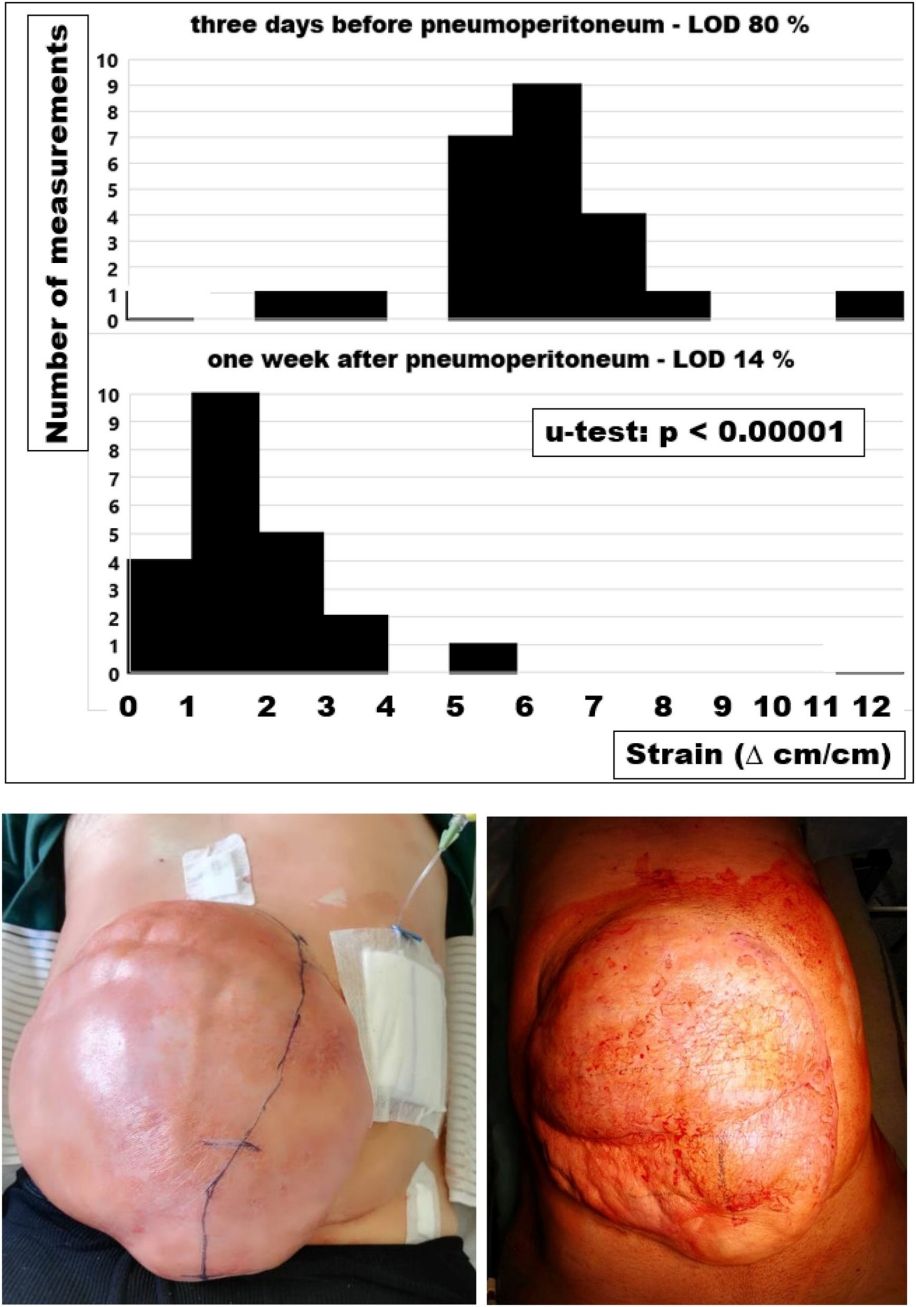
Strain assessed as the regional shift of tissue taken from computed tomography of the abdomen at rest and during a Valsalva maneuver before and after a pneumoperitoneum **(top and middle)** and photos taken during the first filling of the pneumoperitoneum **(bottom left)** and before reconstruction of the abdominal wall **(bottom right)**. The pneumoperitoneum catheter was inserted under laparoscopic control and the incision line was free of adhesions marked under direct vision **(bottom left)**. The tissue distension for three weeks shifts the strain distribution to the left indicating a net gain of peritoneal volume. The strain changes with progressive pneumoperitoneum markedly reduce the loss of domain, ease the shift of the viscera into the peritoneal cavity and decrease the tension on the newly formed abdominal wall after the reconstruction (*u*-test: *p* < 0.00001).

Considering very distensible human tissues, the highest values were found in patients with loss of domain. In a patient with the second recurrence, a laparostoma had led to a loss of domain of 80% (Figure 4). A progressive pneumoperitoneum for 3 weeks distended the abdominal walls, decreased the strain markedly, and reduced the loss of domain to 14%. We concluded that therapeutic effects of preoperative measures such as Botox® injections or progressive pneumoperitoneum could be monitored with computed tomography with Valsalva's maneuver. This approach allowed the biomechanical analysis of the tissue quality in the human situation preoperatively. Knowledge of stiffness, elasticity, distension, or strain permitted the hernia repair to be tailored to the individualized needs of the patient.

### Influence of the Loading Stress

**Figure 5:**
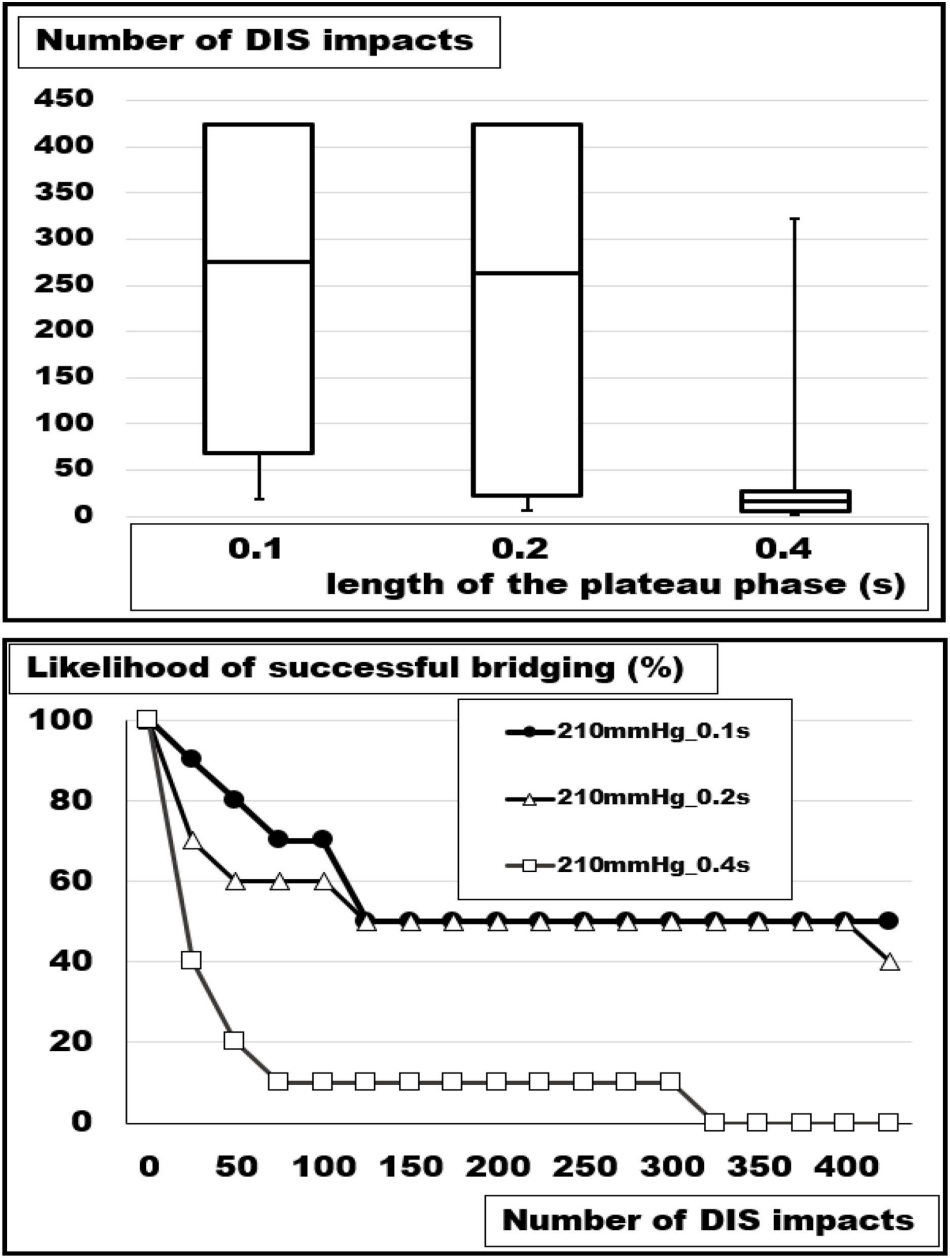
**(Top)** Box- and whisker-plots and **(bottom)** likelihood curves of the number of DIS impacts withstood at plateau lengths of 0.1, 0.2, and 0.4 s. An increasing load destabilizes the reconstruction of a 5 cm orifice bridged with square Dynamesh Cicat® hernia meshes without fixation (Kruskal-Wallis: *p* =0.00852; *u-*test.1 vs.0.4 *p* = 0.00278).

We studied the influence of increasing pulse duration and rising pressure levels using the more elastic bovine flank. Increasing the plateau length from 0.1 to 0.4 s with constant peak pressures at 210 mmHg decreased the likelihood of a successful repair from 50 to 0 % above 300 DIS impacts (Figure 5). Median numbers of DIS pulses withstood dropped significantly from 275 below 25 under these conditions (Kruskal-Wallis test: *p* = 0.00852; *u-*test 0.1 vs. 0.4 s: *p* = 0.00278).

**Figure 6:**
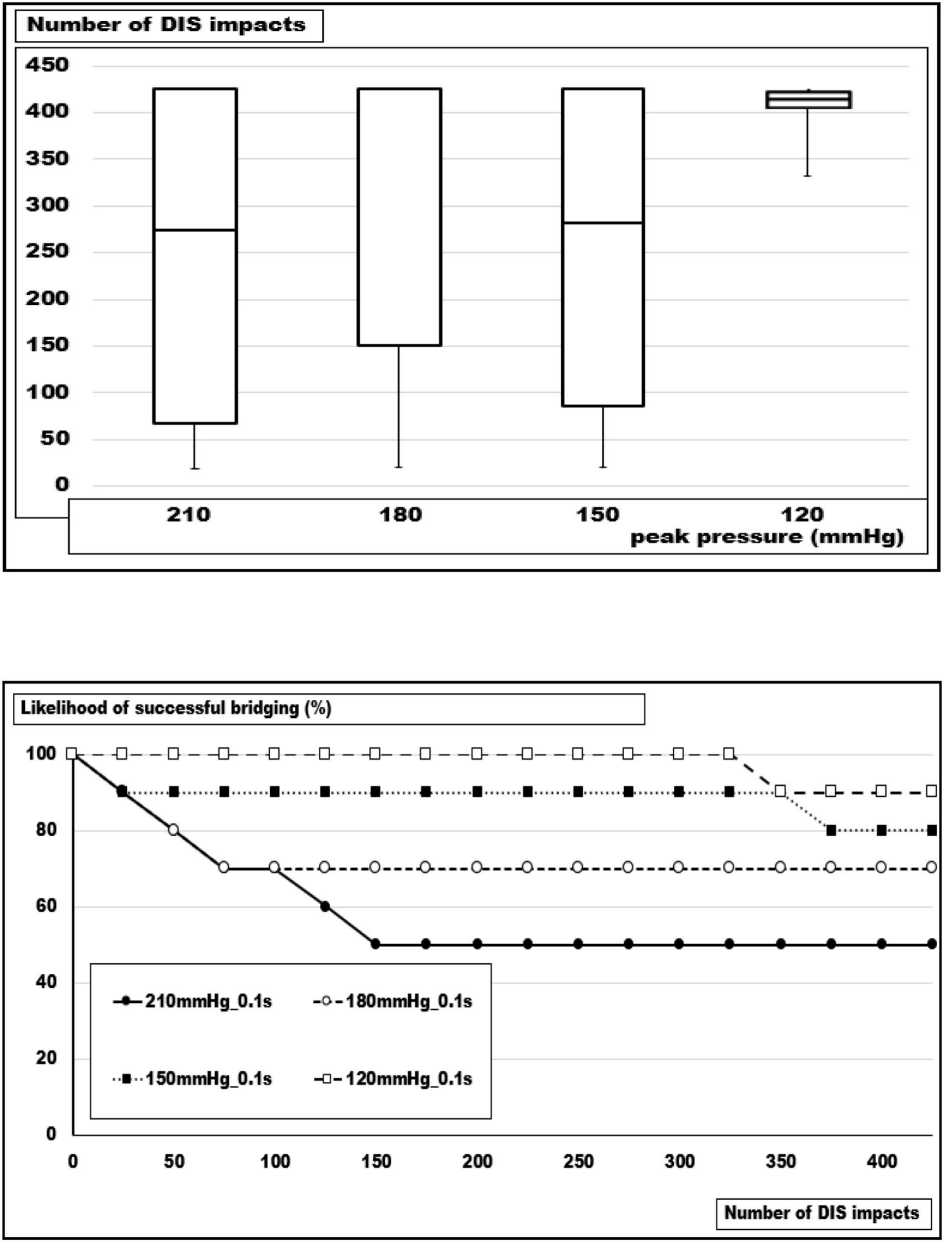
**(Top)** Box- and whisker-plots and **(bottom)** likelihood curves of the number of DIS impacts withstood at pressure peaks of 210, 180, 150, and 120 mmHg. As a trend, an increasing load destabilizes the reconstruction of a 5 cm orifice bridged with square Dynamesh Cicat® hernia meshes without fixation (Kruskal-Wallis: *p* = 0.05705; *u*-test 240 vs. 120 mmHg: *p* = 0.01552).

Increasing the peak pressure from 120 to 210 mmHg at a constant load duration of 100 ms had a destabilizing effect as well but of lower magnitude (Figure 6). After 300 DIS impacts with 120 mmHg peaks, all reconstructions were intact. At this stress level, one repair had failed after another 125 pulses. At 150 and 180 mmHg, an additional 10 and 20% had collapsed at 425 DIS impacts. At this level, one additional repair failed if another 500 pulses were delivered. At loads of 210 mmHg with a plateau length of 0.1 s, 50% of the reconstructions were detached after 150 DIS impacts. Thereafter, no additional slippage was observed. Within this set of data, the differences were not significant (Kruskal-Wallis test: *p* = 0.05705).

### Influence of the Closure Technique and the Resulting Stress Concentrations

**Figure 7:**
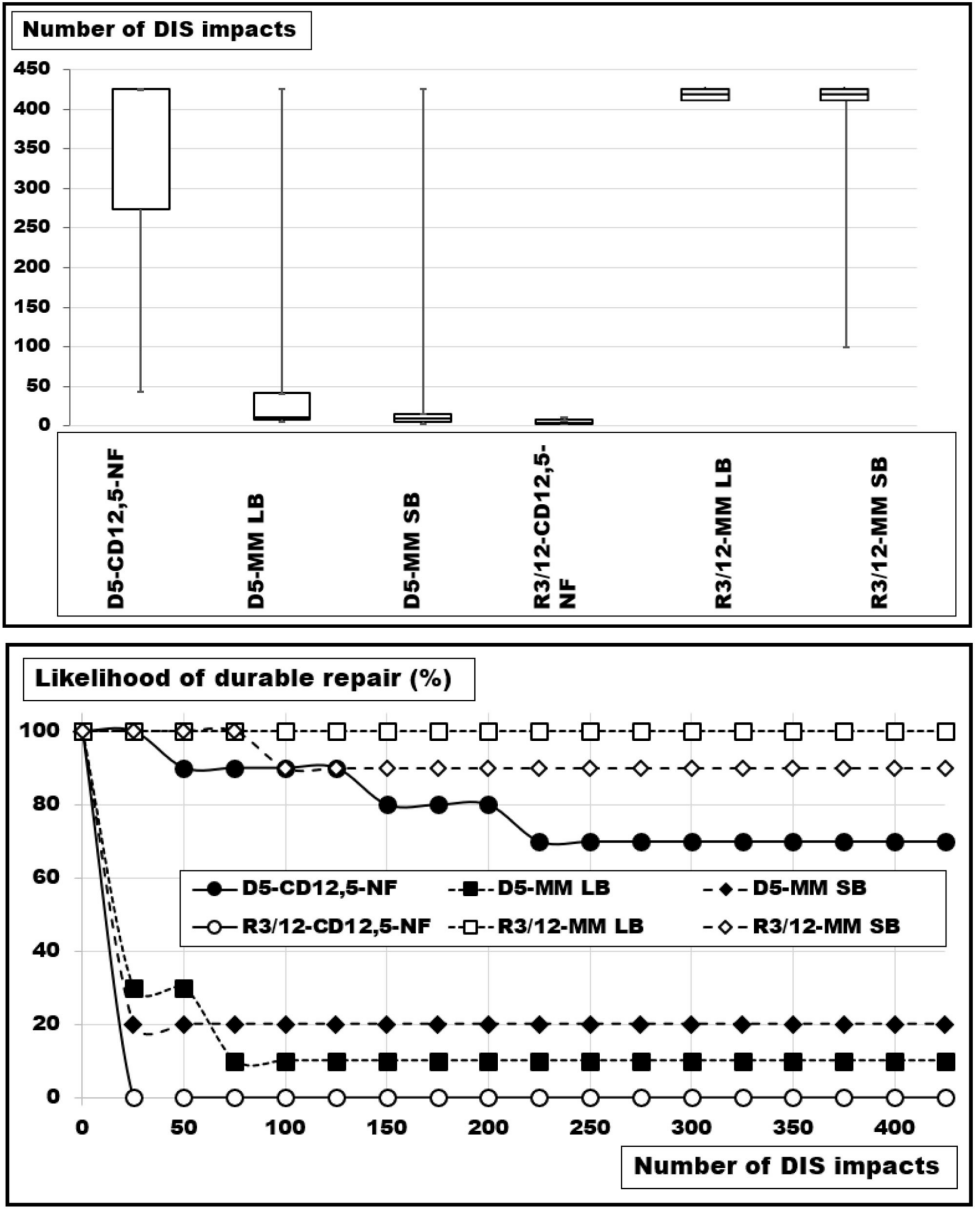
**(Top)** Box-and-whisker-plots and **(bottom)** probability curves of round (D) and rhomboid (R) defects of 20 cm^2^ (D: with a diameter of 5 cm and R: with a long axis of 12.1 cm and a short axis of 3 cm) bridged with a round Dynamesh® Cicat with a diameter of 12.5 cm or closed with 2–0 Monomax® in the large (LB) and the small bite (SB) technique, each with a suture-to-incision ratio above 4:1. The differences are significant (Kruskal-Wallis test: *p* < 0.00005).

Defects of similar sizes, but different shapes were studied using round and rhomboid defects of about 20 cm^2^. The defects were bridged with round Dynamesh® Cicat meshes without fixation or sutured with MonoMax® 2–0 sutures in a large- or a small-bite technique. The likelihood of a durable repair varied between zero and one hundred percent with an expectation of 50% according to previous experiments (7). The results are depicted in more detail in Figure 7.

The round defects were bridged with a probability to last for more than 425 DIS impacts of 70%. Suturing with large bites gave way to nine experiments reliably closing the defect just once (*p* = 0.00194). The small bite technique elevated the safety level by 10% (*p* = 0.00578). The differences between large and small bite- closures were not significant (*p* = 0.65272).

The rhomboid defects were safely closed with sutures. Large bite-sutures were 100 % reliable, small bites only 90% (*p* = 0.72786). Bridging reduced the durability drastically with no reconstruction lasting for more than 15 DIS impacts (*p* = 0.00018). Bridging a rhomboid defect was markedly less stable as compared with a round orifice of the same size (median round: 425 DIS impacts; median rhomboid: 3.5 pulses; *p* = 0.00018). The design used little overlap at the end of the rhomboid shape. The dislocation always occurred here, while not on the sides of the defects.

The circular defects had higher stress concentrations in the middle of the incision but better overlap for the mesh. Therefore, bridging worked better in round defects, and suturing gave better results in the rhomboid orifices with lower stress concentration. The closure techniques for large vs. small bites had an advantage with large bites with more surface providing more retention force. However, the advantage was only about 10%. This difference was small compared with the effects of the stress concentration which made a difference of seventy to ninety percent, respectively, comparing similar closing techniques for hernia defects of similar sizes but of different shapes.

**Figure 8:**
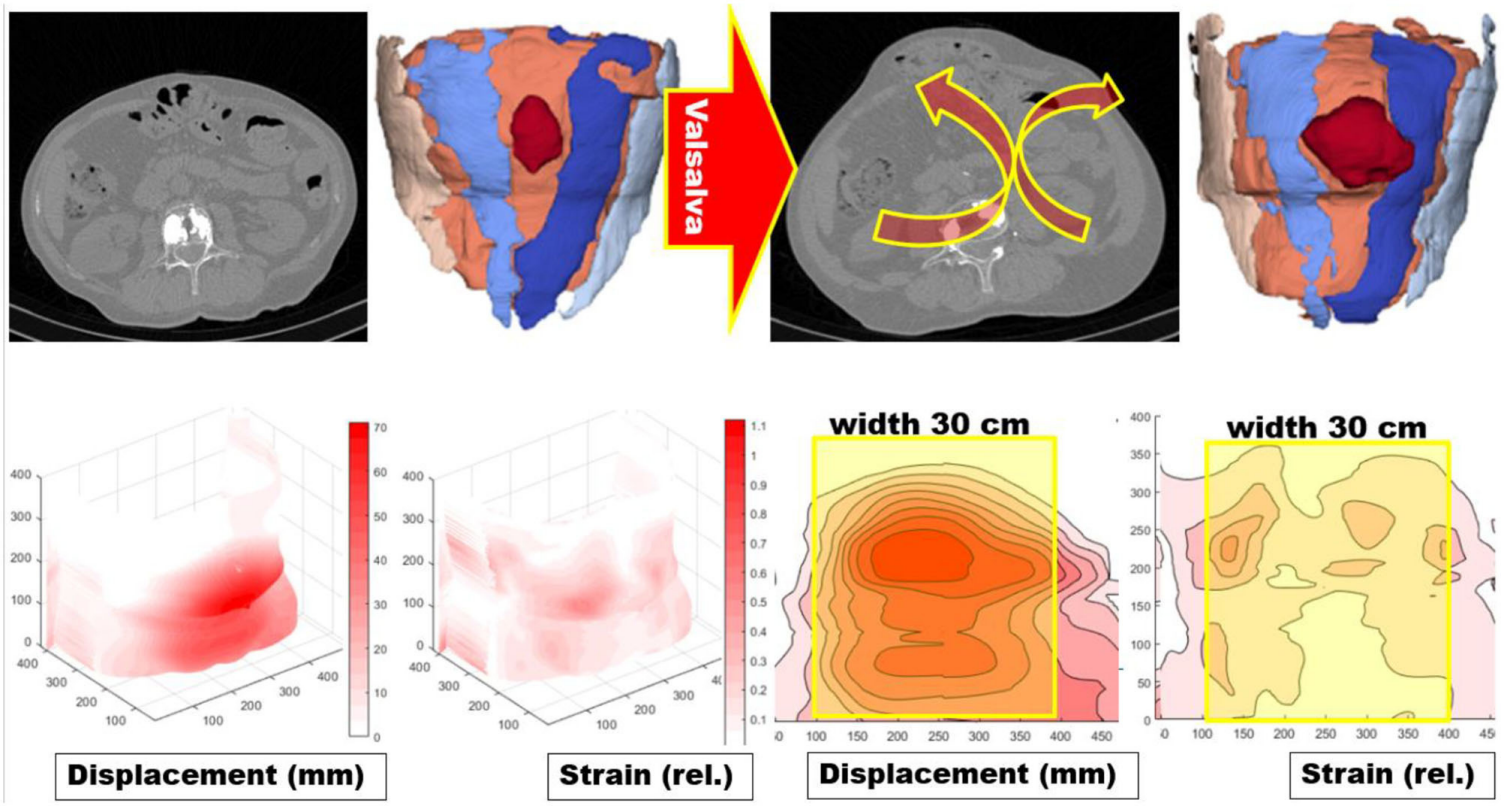
**(Top from left to right)** a scan central to the incisional hernia of a CT abdomen at rest, analysis of the data with artificial intelligence giving the rectus muscles as blue areas and marking the hernia with a dark red color (10). The application of Valsalva's maneuver unmasks a regional instability of the abdominal wall with an outward motion marked with the red arrows with the yellow borders. The resulting hernia orifice enlarges and changes its shape. **(Bottom from left to right)** Surface displacement of the abdominal wall during Valsalva's maneuver in millimeters and the resulting strain field in 3 D and as a planar projection (11). On the planar projections, a hernia mesh 30 cm wide and 45 cm long is superimposed with the center at the umbilicus. It was evident that stress concentrations and peaks of distortions can occur at the edge of the mesh resulting in an unstable reconstruction.

The transfer of these data to the human situation requires the analysis of the spatial strain distribution within the abdominal wall (Figure 8). On average, the hernia area distended by 33% (range: 4–134%). In the individual case, distension varied between 0 and 335%.

This approach permits the analysis of individual peaks of distortion leading to weak spots in the borders of the reconstruction or even outside of the reconstructed area (Figure 8). This is particularly important, when large, complex, or recurrent defects are taken into account. The areas of scars or the peaks of distortions have to be stabilized even if the hernia orifice is some distance away. Distension tends to increase with larger hernia sizes but the correlation is weak (r = 0.64873). Small hernias can already stretch markedly reflecting an elastic tissue quality or an unstable abdominal wall.

**Figure 9:**
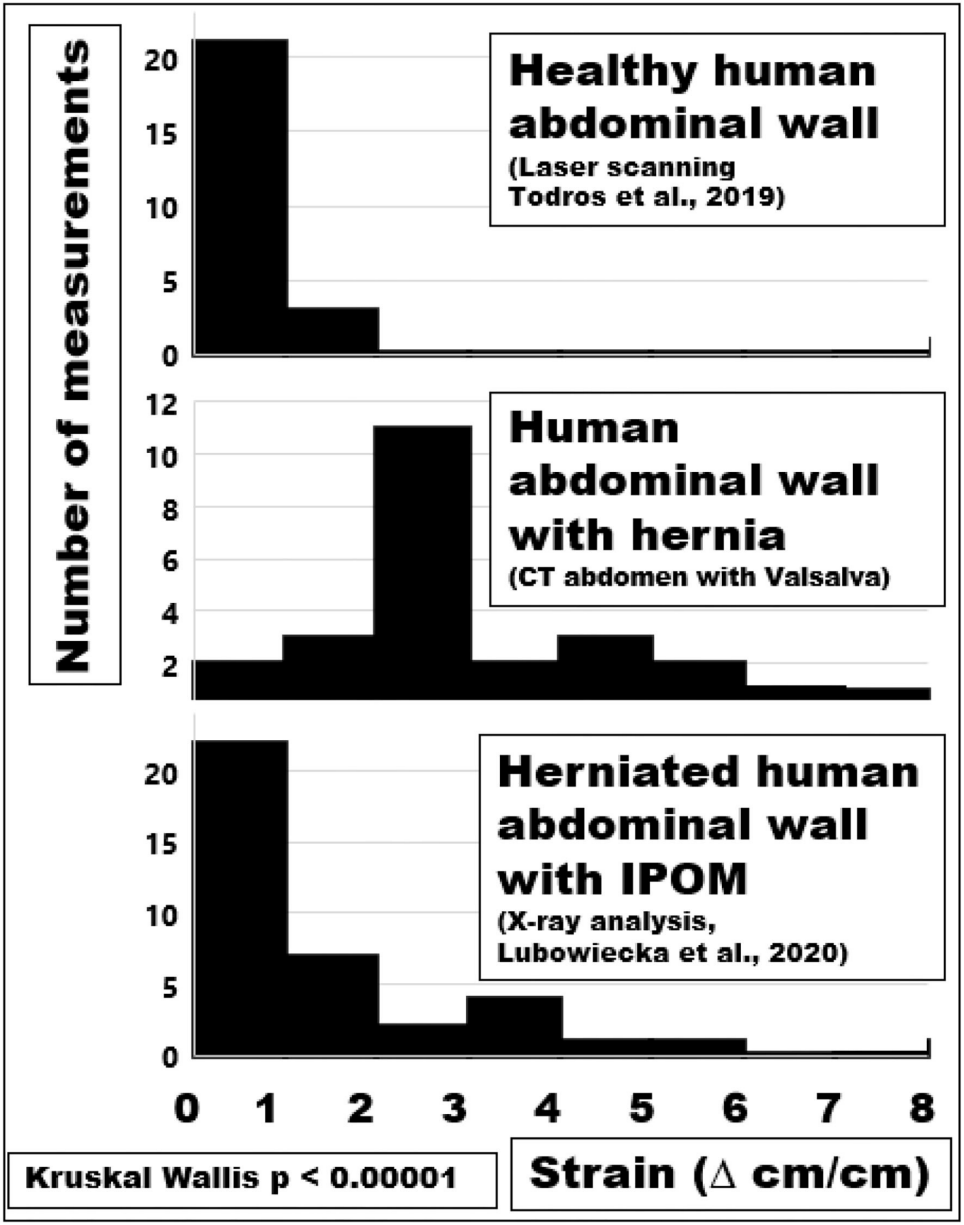
Histograms of the maximum strain distributions of a healthy or herniated abdominal wall compared with values after an IPOM repair **(from top to bottom)**.

From the individual observation, values of shift and distortion could be accumulated as histogram distributions for different groups of patients. The distension of the human abdominal wall with a hernia orifice varied markedly (Figure 9, middle panel). Most herniated abdominal walls had areas that shift several centimeters under pressure. Only 10% of the values fell within the normal range of 1–2 cm. The strain of abdominal walls with a defect was mostly larger. After an IPOM repair, a reduced, but not normalized strain was observed. Since a hernia repair intended to stabilize this tissue shift, the aim was achieved to a certain degree only. Residual strain peaks or contortions could easily overburden a hernia mesh or a fixation element beyond its limits. It was concluded that the hernia mesh and the fixation elements had to be selected to bear the strain levels expected from the preoperative assessment of the individual situation of the patient.

## Discussion

Weak bonds were mechanically overloaded shortly after surgery. The overload resulted in a slackened suture line and occult fascial dehiscence. The invisible dehiscence manifested at a later stage as a recurrent hernia.

The use of textile meshes has been a breakthrough in hernia surgery (6). However, mesh-related complications led to significant unanswered questions for surgeons, patients, hospital systems, and health policymakers throughout the world, having been identified as a “top 10” problem to solve (12). Hernia meshes differ at least 14-fold in adhesiveness, dynamic stiction, and durability to cyclic load (7). Dynamesh® Cicat has a good gripping surface due to its patented basic structure (13). Both lubricants used for the simulation of wet conditions and the attachment properties of tissues can have a variable, but marked influence on the durability of a hernia repair on a bench test using cyclic loading (8). In a clinical registry, durable hernia repair can be designed based on biomechanical principles called the GRIP concept (9, 14). Thus, it remains a question whether mesh-related complications can be prevented considering other entities well known from material sciences of compounds.

### Influence of the Tissue Quality on a Durable Hernia Repair Tested With the Texture Analyser® and With Pulse Loads Delivered by DIS

Surgeons are frequently talking about the tissue quality reflecting the commonly held belief of a well-defined entity. Several terms are used to describe tissue quality, specifically compliance as a measure of distension and elastance as a measure of recoil in hollow vessels or organs. The Young modulus is a measure of the tensile stiffness of a solid organ (6). The data we obtained here are within the range of previously reported values (15). Stress-strain relationships, Young modulus, tensile strength, and load-bearing capacity can be related to the distension of defect-bearing tissues and the durability of a repair to repeated pulse loads (Figures 1, 2).

Soft tissues usually bear the load in the healthy but can weaken with aging, as a gender issue, or as a consequence of medical disease or treatment. Weakening may be associated with a loss of strong collagen fibers (16). Weak soft tissue was a function of the species (porcine vs. bovine), the gender (female vs. male), and of the tissue (skin vs. tendon) from which the collagen was extracted. In this extraction study, the biomechanical properties of the collagen depended on the species and the tissue. In our analysis, stability of the reconstruction also was a function of the strain-bearing capacity of the tissue but depended on the repair technique and the defect size as well (Figure 2). Previously published technical solutions for large hernias such as peritoneal double flaps or a MILOS approach were adapted in the GRIP concept to gain durable hernia repair (9, 14).

### Analysis of Human Tissue Distension *in vivo*

On average, strain-bearing was similar in all tissues investigated (Figure 3). In order to assess the individual levels of different patients, CT scans of the abdomen at rest and during Valsalva's maneuver were found to be helpful. Changes in the tissue quality during a preconditioning period, e.g., with a progressive pneumoperitoneum, were reflected in lower strain levels, in distension of the abdominal cavity, and an increase in useable tissue in repeated CT scans (Figure 4). Since hernia meshes exhibit at least 14-fold variations in the resistance toward repeated impacts, GRIP values should be known and be used as a guide for the reconstruction of the abdominal walls (8). There are no bad meshes but there is not enough knowledge about the creep of meshes upon pulse load (7). We concluded that a surgeon could use bench test data of tissues with different qualities and combine it with a knowledge of the elasticity of the abdominal wall of the individual patient to design a durable hernia repair.

The pressure during Valsalva's maneuver depends on the physical ability and mental willingness of the patient. The strain observed compared almost linearly with the peak pressures in vaginal and rectal tissues (17). It remained unclear whether this holds true for healthy abdominal walls and hernia tissues as well.

### Influence of the Loading Stress

Patients with irritable airways, chronic obstructive lung disease, obesity, or frailty differ from young athletes in the load on the abdominal wall upon coughing, jumping, or exercising with subsequent alterations of the muscular interaction of the pelvic floor, the abdominal wall, the spine, and the diaphragm (18). In order to simulate these influences, we varied the peak pressure and the length of the pulse (Figures 5, 6). We found a marked influence above a peak of 120 mmHg. A doubling of the duration of the pulses destabilized similarly. At this point in time, there is no device to measure and protect the patient postoperatively from these destructive influences (19). An abdominal binder may reduce pain and improve physical function after major abdominal surgery, but its role after open incisional hernia surgery remains controversial (20, 21). The role of anesthesia should be highlighted within the context of perioperative minimization of coughing with a laryngeal mask seemingly being superior to intubation (22).

Surgeons observed for decades that aponeurosis approximated by sutures can slacken and open the road to hernia formation (3). Higher loading stress will increase the risk. Recurrence indicates a bond between tissue and mesh too weak to withstand physical activity durably. The weak bond is mechanically overloaded early (1). Healing is impaired since non-crosslinked collagen stretches and results in occult fascial dehiscence (2). A continuously overburdened healing process manifests at a later stage as a recurrent hernia defect in humans and experimental animals (3, 4). Monitoring of such unwanted developments requires the integration of strain sensors in hernia meshes, at least in high-risk groups (23). Recent developments permit the monitoring of strain on hernia meshes upon the physical activities of the patient (24).

Prevention of unwanted recurrence at the time of surgery is our approach to a durable repair. In the Grip concept, the minimally required retention force necessary for the reconstruction to withstand expected strain levels is calculated. During the preoperative planning, a durable repair is designed. Intraoperatively, the design can be adjusted to notches, weak or unstable areas, and other findings. This approach improved clinical results (9, 14). With this approach, expected higher loading stress can be accounted for adding a safety margin to the repair.

### Influence of the Closure Technique and the Resulting Stress Concentrations

Closure of hernia defects with similar sizes but different shapes should need comparable GRIP values for a durable repair (25, 30). In contrast, varying shapes require different techniques to resist cyclic loading (Figure 7). The load-bearing capacity of the reconstruction seems to vary according to the load-bearing capacity of the curved edge of the defects. Such stress concentrations around an empty hole increase the maximum stress at the edge of a hole by a factor of three [Stress Concentrations at Holes (https://www.fracturemechanics.org/hole.html)]. *In vivo*, tissue responses increase the collagen content in “open” wounds after 6–9 days (26). During the first days after defect closure, a slackening of the suture line can occur due to stress concentrations related to the defect shapes. In poor quality tissues, hernia formation is a consequence of repeated overstretching of the newly formed collagen (2). Before a hernia is formed a debris zone of an unstable abdominal wall occurs which can be detected with a CT abdomen at rest and during Valsalva's maneuver (Figure 8). This is particularly important in a battlefield abdomen with multiple scars. The mesh must cover all unstable areas widely otherwise giving way to pseudo-recurrences at the edge or in distant unstable regions.

The overlap depends on the area available, the mechanical properties of polymers, and the second moment of inertia (https://en.wikipedia.org/wiki/Second_moment_of_area). For this problem, no theoretical solution is available yet for a mesh-tissue combination but experimentally, the overlap can be reduced as the mesh coefficient increases (7).

A herniated abdominal wall exhibits several-fold increases in strain compared with healthy ones (Figure 9). The reinforcement of a herniated abdomen with an IPOM mesh reduced the strain without normalizing it (27). Since commercially available hernia meshes markedly differ in their biomechanical properties the design of a hernia repair should aim above the critical resistance to impacts related to pressure (6, 9, 14).

The residual strain after a reconstruction burdens the herniated abdomen potentially life-long but muscle fibers reinforced with mesh can adapt over time (28). Nevertheless, measures should be taken to minimize the persistent mechanical stress of an incised or herniated abdomen for a durable repair (29). Such measures are described in the GRIP concept above. Clinically, a cohort of patients was treated accordingly and reported in this journal with excellent results (Nessel et al., 2021, in press). We concluded that mesh-related complications, such as hernia recurrence, could be reduced considering biomechanical properties of the meshes, fixation-related influences, and tissue properties.

## Data Availability Statement

The raw data supporting the conclusions of this article will be made available by the authors, without undue reservation.

## Ethics Statement

The use of the tissues was permitted by Bürgeramt Veterinärwesen der Stadt Heidelberg according to European law with the permission DE 08 221 1018 21. The studies involving human participants were reviewed and approved by the Ethics Committee of the Heidelberg University vote S-522/2020. The patients/participants provided their written informed consent to participate in this study.

## Author Contributions

FK, VH, JG, and RN designed the research, conducted some of the experiments, evaluated the results and drafted the manuscript. YL, DG, CG, HS-H, LK, CL, KU, PL, SV, and MV conducted some of the experiments, evaluated the results and drafted the manuscript. All authors contributed to the article and approved the submitted version.

## Funding

Heidelberger Stiftung Chirurgie Grants Nos. 2016/22, 2017/171, 2018/215, 2019/288, 2020/376, and 2021/444 for the research described in the manuscript, financial support only.

## Conflict of Interest

FK has received research grants from Baxter^®^, Dahlhausen^®^, and Medtronic^®^ not related to the research perspective described in the manuscript. The remaining authors declare that the research was conducted in the absence of any commercial or financial relationships that could be construed as a potential conflict of interest.

## Publisher's Note

All claims expressed in this article are solely those of the authors and do not necessarily represent those of their affiliated organizations, or those of the publisher, the editors and the reviewers. Any product that may be evaluated in this article, or claim that may be made by its manufacturer, is not guaranteed or endorsed by the publisher.
